# Levels of neuropeptide Y in synovial fluid relate to pain in patients with knee osteoarthritis

**DOI:** 10.1186/1471-2474-15-319

**Published:** 2014-09-27

**Authors:** Lei Wang, Li Zhang, Haobo Pan, Songlin Peng, Minmin Lv, William Weijia Lu

**Affiliations:** Center for Human Tissues and Organs Degeneration, Shenzhen Institute of Advanced Technology, Chinese Academy of Science, 1068 Xueyuan Avenue, 518055 Shenzhen, China; Department of Orthopedic Surgery, People’s Hospital of Hangzhou, Nanjing Medical University, 261 Huansha Road, Hangzhou, 310006 China; Institute of Biomedicine and Biotechnology, Shenzhen Institute of Advanced Technology, Chinese Academy of Science, 1068 Xueyuan Avenue, Shenzhen, 518055 China; Department of Orthopedic Surgery, People’s Hospital of Shenzhen, Jinan University Second College of Medicine, 1017 Dongmen North Road, Shenzhen, 518020 China; Department of Orthopaedics and Traumatology, Li Ka Shing Faculty of Medicine, The University of Hong Kong, Pokfulam, China

**Keywords:** Pathogenesis, Arthrophlogosis, Synovia, Radioimmunoassay, Regulator

## Abstract

**Background:**

The precise etiology of knee osteoarthritis (KOA) pain remains highly controversial and there is no known effective treatment. Due to the known and suggested effects of neuropeptide Y (NPY) on pain, we have sought to investigate the relationship between the concentration of NPY in synovial fluid of knee, pain of KOA, and structural severity of KOA.

**Methods:**

One hundred KOA patients and twenty healthy participants (control group) were recruited. The pain and the radiographic grade of KOA were assessed separately by Hideo Watanabe’s pain score and Tomihisa Koshino’s scoring system. Synovial fluid of knee from all participants was collected with arthrocentesis. Radioimmunoassay was used to examine the concentration of NPY in synovial fluid of knee.

**Results:**

Concentrations of NPY in synovial fluid were significantly higher in KOA patients (124.7 ± 33.4 pg/mL) compared with controls (64.8 ± 26.3 pg/mL) (*p* = 0.0297). According to Hideo Watanabe’s pain score, 100 KOA patients were divided into 5 subgroups: no pain (n = 12), mild pain (n = 25), moderate pain (n = 37), strong pain (n = 19) and severe pain (n = 7). Within the KOA group, significantly higher concentrations of NPY were found in each subgroup as pain intensified (no pain 81.4 ± 11.7 pg/mL, mild pain 99.1 ± 23.2 pg/mL, moderate pain 119.9 ± 31.5 pg/mL, strong pain 171.2 ± 37.3 pg/mL and severe pain 197.3 ± 41.9 pg/mL). Meanwhile, according to Tomihisa Koshino’s scoring system, 100 KOA patients were divided into 3 subgroups: early stage (n = 30), middle stage (n = 53), advanced stage (n = 17). Concentrations of NPY in middle and advanced stage groups of KOA patients were significant higher than early stage group of KOA patients (early stage 96.4 ± 27.1 pg/mL, middle stage 153.3 ± 16.9 pg/mL, advanced stage 149.5 ± 36.7 pg/mL) (*p =* 0.0163, *p* = 0.0352). Concentrations of NPY in advanced stage group of KOA patients has no significant difference compare with middle stage group of KOA patients (*p =* 0. 2175).

**Conclusions:**

This study demonstrated the presence and variation of concentrations of NPY in the KOA joint fluid, suggesting a role for NPY as a putative regulator of pain transmission and perception in KOA pain.

**Electronic supplementary material:**

The online version of this article (doi:10.1186/1471-2474-15-319) contains supplementary material, which is available to authorized users.

## Background

Knee osteoarthritis (KOA) is a chronic degenerative joint disorder that affects a large proportion of the population, particularly in elderly people [1–6]. Epidemiological studies have revealed that over 70% of people aged 65 years or older suffer from OA with the knee joint being most commonly affected [7]. KOA patients’ major clinical manifestation is chronic pain that typically worsens as a result of weight bearing, activity or movement of the affected joint [8]. Synovial inflammation raised intra-osseous pressure and mechanical stresses on intra-articular and peri-articular ligaments and tendons [9–11] are potential contributors to the chronic pain encountered. However, the precise aetiology of KOA pain remains highly controversial, which limits the progress in developing effective treatments for KOA pain [12].

Neuropeptide Y (NPY), a 36 amino acid peptide, is one of the most widely distributed neuropeptides in the nervous system [13, 14]. It has diverse and complex biological functions, such as capacity to influence cardiovascular performance, food intake and pain processing [15–17]. In addition, pathophysiological role of NPY in infection and inflammation, as well as in autoimmunity, has been suggested [18, 19]. The up-regulation of NPY in the dorsal root ganglia and spinal cord has been shown in various models of inflammatory and neuropathic pain [20–23] and around blood vessels in the capsule of the joint [24–26]. NPY and its Y1 and Y2 receptors are located at key pain signaling centers throughout the nervous systems [27–32]. Previous work also suggested that joint pain results from the activation of primary afferent nerve fibers by neuropeptides at the joint [33–35]. Due to the known and suggested important effects of NPY on pain, we hypothesized that NPY may be involved in the pathogenesis of KOA pain. Therefore, the aim of this study was to assess the relationship between concentrations of NPY in the synovial fluid of knee, KOA pain, and structural severity of KOA.

## Methods

### Ethics statement

This study is in accordance with the ethical principles of the Declaration of Helsinki and was approved by the Local Research Ethics Committee (Research Ethics Committee of People’s Hospital of Hangzhou, Nanjing Medical University), and the trial number 023–01 from Research Ethics Committee of People’s Hospital of Hangzhou, Nanjing Medical University.

All participants provided informed, written consent.

### Patients

This one year study was conducted in People’s Hospital of Hangzhou, Nanjing Medical University from January 2009 to January 2010.

One hundred KOA patients were recruited from the department of orthopedic surgery in People’s Hospital of Hangzhou, fulfilling the American College of Rheumatology clinical criteria for the diagnosis of KOA [36]. In case of bilateral KOA, the more serious pain and/or edema and/or deformed side (determined by the patient’s subjective judgment) was assessed. Exclusion criteria included knee joint trauma ever in their lives (periarticular fracture, meniscectomy, etc.), other arthritis (gout, rheumatoid arthritis, purulent arthritis, etc.), metabolic bone diseases (osteoporosis, Paget’s disease, osteopetrosis, etc.), malignancy, bone tumor (multiple myeloma, etc.), primary or secondary hyperparathyroidism, inflammatory arthropathy and any knee surgery during the last 6 months. In addition, patients were excluded if any anti-inflammatory drugs (oral NSAID, etc.), odynolysis and/or cortico-therapy were used within the past 4 weeks.

Twenty healthy participants between 35 and 65 years of age without any diseases judged by the physician were recruited from the People’s Hospital of Hangzhou as the control groups.

### KOA pain assessment

Pain was assessed by the physician based on the patient’s medical history according to Hideo Watanabe’s knee scoring system-related pain score [37]. Patients with KOA were divided into 5 groups: no pain group, mild pain group, moderate pain group, strong pain group and severe pain group (Table 1).

**Table 1:** **Hideo Watanabe’s knee scoring system-related pain score**

Group	Standard
No pain group	Occasionally feeling fatigue or heaviness, but no pain at any time
Mild pain group	Pain at starting time of various activities or occasionally during long-distance walking, but no pain at rest
Moderate pain group	Pain usually on walking, but pain gradually subside after a brief rest
Strong pain group	Persistent pain on walking, but pain gradually mitigates after a rest, usually associate with spontaneous pain
Severe pain group	Persistent pain at any time, including walking and rest

### KOA radiographic grade

Full-extension posterior-anterior radiographs (X-ray) of the knees were obtained and assessed by the physician. The degree of radiographic KOA in individual joints was graded (0 to 5) by the study investigator using the Tomihisa Koshino’s scoring system [38]. Grade 1 was considered early stage, grade 2–3 was middle stage and grade 4–5 was advanced stage (Table 2).

**Table 2:** **Tomihisa Koshino’s radiographic grading for osteoarthritic knees in a weightbearing position** ^**a**^

Stage	Grade	Standing x-ray
	0	Normal
Early stage	1	Bone sclerosis or osteophyte formation
Middle stage	2	Narrowing of joint space (≤3 mm)
	3	Obliteration of joint space or subluxation^b^
Advanced stage	4	Defect of tibial plateau (<5 mm)
	5	Defect of tibial plateau (≥5 mm)

^a^An anteroposterior and weight-bearing radiograph taken in a standing position was used for grading.

^b^“Subluxation” indicates the condition in which the medial edge of the medial tibial plateau shows a lateral shift by more than 5 mm against the medial edge of articular surface of the medial femoral condyle without including osteophyte.

### Arthrocentesis & joint fluid sampling

All participants were in a supine position on a stretcher. The same entry site was demarcated with a skin-marking pen. The skin was prepared with povidone-iodine. A sterile drape was placed around the site. Then the region was anesthetized by placing a wheal of lidocaine, using a small (25-gauge) needle. Intermittently the plunger was pulled back during the injection of the anesthetic to exclude intravascular placement.

An 18-gauge needle was used directly behind the patella into the synovial cavity with the lateral approach. Upon insertion into the articular cavity, 3 mL of 0.9% saline was injected slowly into the joint and after 20s 3mL of turbid-appearing fluid was aspirated and immediately centrifuged (2000 rev/min, 10 min) at 4°C and stored at -70°C until analyzed. Synovial fluid from healthy individuals were collected, frozen, and stored in the same way as the KOA patients.

### NPY in synovial fluid

Radio-immunoassay was performed to determine concentrations of NPY in KOA synovial fluid. Concentrations of NPY in joint fluid was determined by commercially available radioimmunoassay kits (Iodine [^125^I] Neuropeptide Radioimmunoassay kit, Institute of RIA, Chinese PLA General Hospital, China) in accordance with the standard protocols included in the kits. The sensitivity of the radio-immunoassay kit was < 33 pg/mL.

### Statistical analysis

All analyses were performed using SPSS version 13.0. Data presented as mean ± SD. Bartlett’s method was performed first to identify the homogeneity of variances. Group *t*-test was used to compare the mean of concentrations of NPY in synovial fluid of KOA Group with Healthy control group. Dunnett-*t* test was used to compare the mean of concentrations of NPY in synovial fluid of each subgroup of KOA Group with Healthy control group. In addition, the Student-Newman-Keuls (SNK) test was used to compare the mean of concentrations of NPY in synovial fluid of each KOA subgroup. A linear regression was used to assess the relationship between concentrations of NPY in synovial fluid and the pain of KOA patients. A *p* value less than 0.05 was considered to be statistically significant.

## Results

### Participant demographics

In total, 100 KOA patients and 20 healthy controls participated in this study. The mean age of the KOA patients was 56 ± 6.9 years and 48 ± 8.1 years for the healthy controls, which was not significantly different (*p* = 0.347). The gender distribution between the two groups was similar with 39% of the KOA patients and 50% of healthy controls being male (*p* = 0.3609).

### KOA pain and NPY concentrations

**Figure 1:**
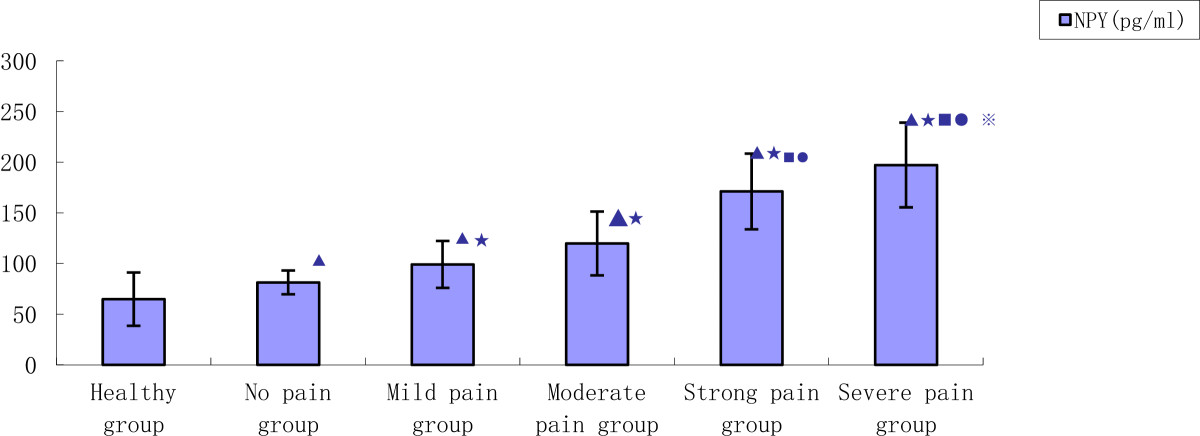
**Comparison of pain and NPY concentrations of KOA patients with healthy participants.** (*p* < 0.05: ▲ vs. Healthy control group; Intragroup KOA ^★^vs. No pain group, ^■^vs. Mild pain group, ^●^vs. Moderate pain group, ^※^vs. Strong pain group).

**Figure 2:**
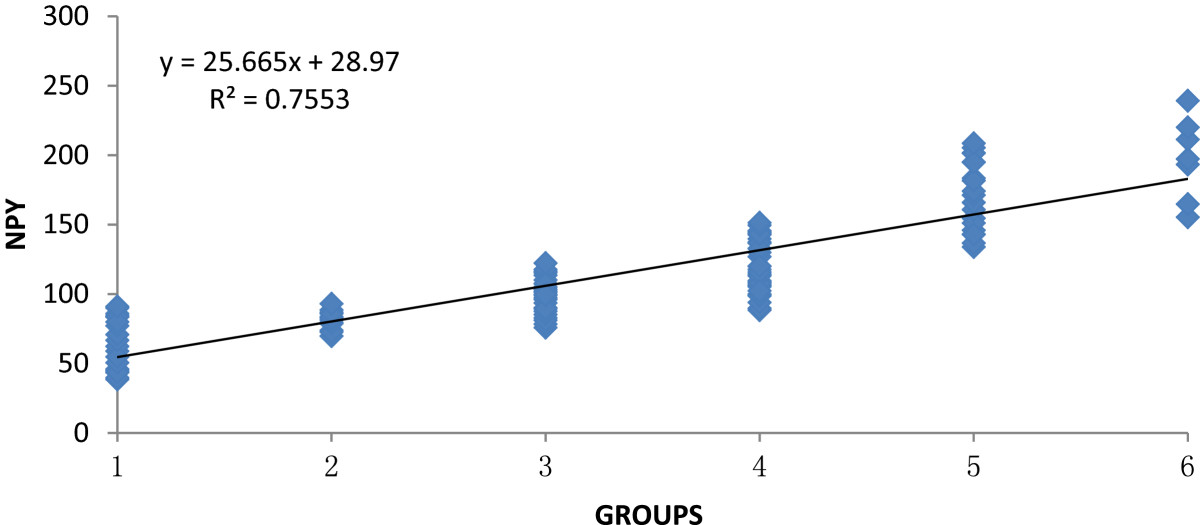
**The correlation between synovial fluid NPY concentration and pain of KOA patients.** (1 = Healthy control group, 2 = No pain group, 3 = Mild pain group, 4 = Moderate pain group, 5 = Strong pain group, 6 = Severe pain group).

As previously stated the KOA patients (n = 100) were divided into 5 groups according to Hideo Watanabe’s pain score: no pain group (n = 12), mild pain group (n = 25), moderate pain group (n = 37), strong pain group (n = 19) and severe pain group (n = 7) (Figures 1 and 2).

In all tested KOA groups, there was a positive correlation between concentrations of NPY and the level of pain. Concentrations of NPY were significantly higher in KOA patients (124.7 ± 33.4 pg/mL) than in healthy participants (64.8 ± 26.3 pg/mL) (*p* = 0.0297). Within KOA subgroups, significantly higher concentrations of NPY were found in each subgroup as pain increased (no pain group 81.4 ± 11.7 pg/mL, mild pain group 99.1 ± 23.2 pg/mL, moderate pain group 119.9 ± 31.5 pg/mL, strong pain group 171.2 ± 37.3 pg/mL and severe pain group 197.3 ± 41.9 pg/mL).

### KOA radiographic grade and NPY concentrations

**Figure 3:**
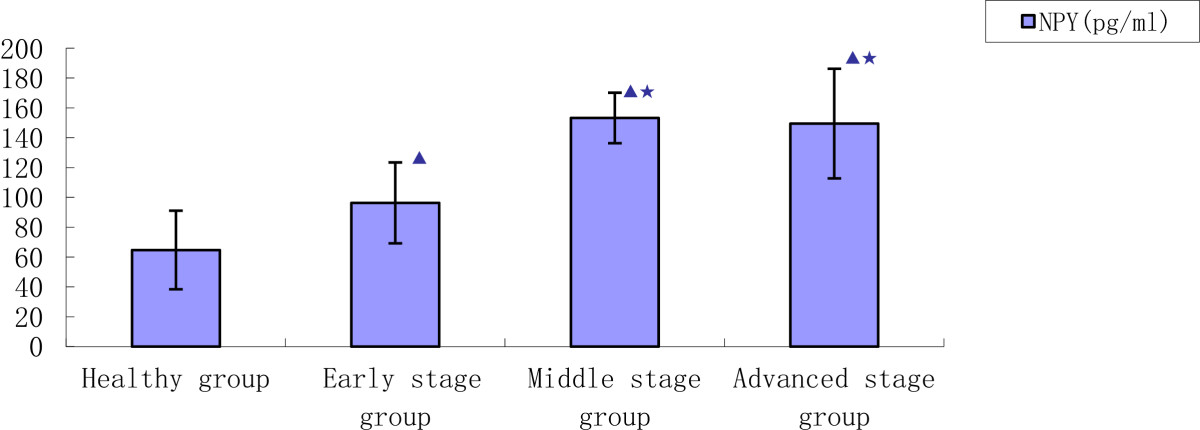
**Comparison of radiographic grade and concentrations of NPY of KOA patients with healthy participants.** (*p* < 0.05: ▲ vs. Healthy control group; Intragroup KOA ^★^vs. Early stage group).

As previously stated, 100 KOA patients were divided into 3 stage groups according to Tomihisa Koshino’s scoring system: early (n = 30), middle (n = 53) and advanced (n = 17) (Figure 3).

Concentrations of NPY were significantly higher in the KOA patients (124.7 ± 33.4 pg/mL) compared to the healthy controls (64.8 ± 26.3 pg/mL) (*p =* 0.0297). Concentrations of NPY in the middle and advanced staged patients (153.3 ± 16.9 pg/mL, 149.5 ± 36.7 pg/mL) were significantly higher compared to the early staged patients (96.4 ± 27.1 pg/mL) (*p =* 0.0163, *p* = 0.0352). Concentrations of NPY in the advanced staged patients were not significantly different compared to the middle staged patients (*p =* 0. 2175).

## Discussion

Despite the widespread prevalence of KOA in the adult population, very little is known about the source of KOA pain, which may be initiated by chemical mediators in KOA joints [39]. Due to the role of NPY in pain [20–23, 27–32], we sought to study the effect of varying concentrations of NPY in KOA joint fluid and its association with the pain, and structural severity of KOA. To our knowledge, this is the first study specifically designed to evaluate the relationship between concentrations of NPY, KOA pain, and structural severity of KOA.

Our primary finding was that concentrations of NPY were significantly higher in KOA patients compared to healthy controls. The results of our study were in agreement with the available clinical literature, which has also found significantly higher concentrations of NPY in the synovial fluid of patients with arthritis of the knee (crystal induced arthritis, chronic polyarthritis, post-infectious arthritis, rheumatoid arthritis), compared to control patients with non-inflammatory joint disorders (lateral meniscus injury, medial meniscus injury, cruciate ligament injury), admitted for arthroscopy [40, 41].

With an increase in pain based on each KOA subgroups we found significantly higher concentrations of NPY. This result was similar to literatures that found that pain gradually developed from the initial mild pain into a long period of severe pain during the pathological process of KOA [9–12]. This indicates that levels of NPY are related to the joint pain in patients with KOA.

Concentrations of NPY in synovial fluid of middle and advanced KOA stages were significantly higher than early KOA stage. But concentrations of NPY in synovial fluid of advanced stage of KOA patients have no significant difference to compare with middle stage of KOA patients. These results contradict the notion that NPY has significant relevance to joint pain in patients with KOA, and significantly higher concentrations of NPY may lead to a significant increase in pain. It suggests a lack of agreement between X-rays evidence of KOA and patients’ report of pain at that site base on the result of our studies, which are the first study specifically designed to evaluate the relationship between NPY and KOA pain. Meanwhile, the orthopedic community has been plagued for years by this discordance. Many researchers [42–46] have found evidence for a substantial discordance between pain and observed radiographic evidence of KOA. In a 2008 systematic review of population studies, Bedson and Croft quantitatively described the problem for KOA: “In those with radiographic KOA the proportion with pain ranged from 15% to 81%” [47]. The discordance between pain and radiographic KOA points to the need for further investigation of this phenomenon. And, the different sub-scales of classifications for grade of KOA may be one of the causes. The obliteration of joint space, which is considered middle stage in this study, is quite advanced KOA and according to the Kellgren-Lawrence scoring system would represent end-stage KOA (grade 4). This may help explain why no differences were found between middle and advanced KOA groups.

Studies have shown that during arthritis, pro-inflammatory mediators are released into the joint [48] which sensitize the joint afferent neurons. This can cause previous innocuous physicochemical stimuli to activate these neurons which will lead to the sensation of joint pain [49, 50]. One important family of agents known to be involved in the peripheral sensitization of joint afferents is the inflammatory neuropeptides, which include NPY [30–32, 51]. NPY (belonging to the pancreatic polypeptide family) was first isolated from pig brain by Tatemoto [24]. NPY is produced together with noradrenaline in certain sympathetic nerve fibers [25] and has a strong and long-standing vasoconstrictive effect on both arterial and venous vessels. In the rat, this neuropeptide was found around blood vessels in the capsule of the joint, but not in the disc or cartilaginous joint surfaces [26]. This potent neuromodulator is stored in the terminal branches of Aδ and C fibers where it’s release into the joint lowers the activation threshold of nociceptive nerve endings, which is likely to contribute to chronic, sensitized pain responses [52]. Based on the current study, the presence and variation of NPY in KOA joint fluid strongly points to a role as a regulator of pain transmission and perception in KOA pain. Possible mechanisms by which NPY can modulate pain processing. NPY can lower membrane Ca^2+^ conductance in dorsal root ganglion neurons and inhibits substance P released from the central terminals of the primary afferent fibers [14, 53, 54]. Furthermore, the observation that peripheral inflammation increases both NPY and its Y1 and Y2 receptor synthesis in the spinal dorsal horn reinforces the concept that spinal NPY participates in the processing of nociception [12]. Noradrenergic neurons of the locus coeruleus and A1 noradrenergic cell groups also constitute a major system concerned with the modulation of nociception [55] and NPY is co-localized with noradrenaline in a sub-population of the neurons [25, 56, 57]. In the locus coeruleus, NPY depresses the spontaneous firing rate of these neurons and the hyperpolarizing effect of α_2_-agonists through stimulation of its Y2 receptor subtype [58].

### Study limitations

This study is limited by a small sample size and the different sub-scales of classifications for pain and grade of KOA. In addition, the underlying molecular and cellular mechanisms of NPY in KOA pain remain poorly understood. Hopefully, future studies will provide answers to these questions.

## Conclusions

This study demonstrated the concentrations of NPY between KOA patients and healthy controls. These results suggest a role for NPY as a putative regulator of pain transmission and perception of KOA pain. In addition, concentrations of NPY may reflect the pathological progressing and severity of KOA. The precise roles of NPY in the pathogenesis of KOA pain require further investigation. However, our results have contributed to a better understanding of the molecular processes underlying KOA pain and, in addition, foster the option of local therapeutic intervention targeting NPY. The understanding of the role of NPY in KOA pain is a prerequisite to developing such novel therapeutic options for the treatment of KOA pain and restoration of tissue function.

## Authors’ original submitted files for images

Below are the links to the authors’ original submitted files for images. Authors’ original file for figure 1 Authors’ original file for figure 2 Authors’ original file for figure 3

## Abbreviations

KOA: Knee osteoarthritis
OA: Osteoarthritis
NPY: Neuropeptide Y
NSAID: Non-steroidal anti-inflammatory drug
RIA: Radioimmunoassay
SPSS: Statistical Product and Service Solutions
SNK test: Student-Newman-Keuls test.
