# Supplementary figures and images for: Levels of neuropeptide Y in synovial fluid relate to pain in patients with knee osteoarthritis
Source: BMC Musculoskelet Disord. 2014 Sep 27;15:319. doi: 10.1186/1471-2474-15-319 (PMC4195915; doi:10.1186/1471-2474-15-319)

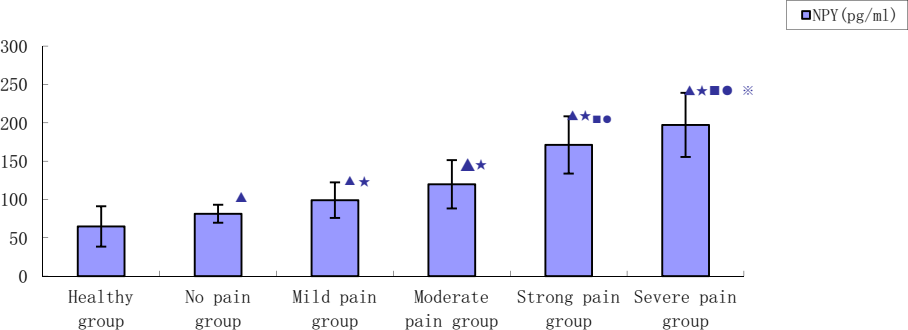

Supplement: Supplementary file 1 — Authors’ original file for figure 1 [file 12891_2014_2268_MOESM1_ESM.pdf]

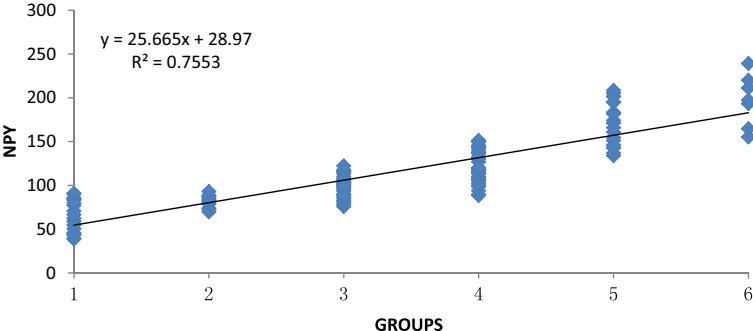

Supplement: Supplementary file 2 — Authors’ original file for figure 2 [file 12891_2014_2268_MOESM2_ESM.pdf]

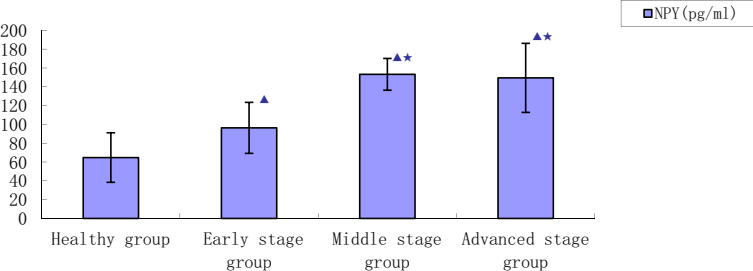

Supplement: Supplementary file 3 — Authors’ original file for figure 3 [file 12891_2014_2268_MOESM3_ESM.pdf]
